# Post-injury ventricular enlargement associates with iron in choroid plexus but not with seizure susceptibility nor lesion atrophy—6-month MRI follow-up after experimental traumatic brain injury

**DOI:** 10.1007/s00429-021-02395-5

**Published:** 2021-11-10

**Authors:** Amna Yasmin, Asla Pitkänen, Pedro Andrade, Tomi Paananen, Olli Gröhn, Riikka Immonen

**Affiliations:** grid.9668.1 0000 0001 0726 2490 A.I.Virtanen Institute for Molecular Sciences, University of Eastern Finland, 1627, Kuopio, Finland

**Keywords:** Brain–CSF barrier, Epileptogenesis, Heme, Idiopathic normal pressure hydrocephalus, Ventriculomegaly

## Abstract

Ventricular enlargement is one long-term consequence of a traumatic brain injury, and a risk factor for memory disorders and epilepsy. One underlying mechanisms of the chronic ventricular enlargement is disturbed cerebrospinal-fluid secretion or absorption by choroid plexus. We set out to characterize the different aspects of ventricular enlargement in lateral fluid percussion injury (FPI) rat model by magnetic resonance imaging (MRI) and discovered choroid plexus injury in rats that later developed hydrocephalus. We followed the brain pathology progression for 6 months and studied how the ventricular growth was associated with the choroid plexus injury, cortical lesion expansion, hemorrhagic load or blood perfusion deficits. We correlated MRI findings with the seizure susceptibility in pentylenetetrazol challenge and memory function in Morris water-maze. Choroid plexus injury was validated by ferric iron (Prussian blue) and cytoarchitecture (Nissl) stainings. We discovered choroid plexus injury that accumulates iron in 90% of FPI rats by MRI. The amount of the choroid plexus iron remained unaltered 1-, 3- and 6-month post-injury. During this time, the ventricles kept on growing bilaterally. Ventricular growth did not depend on the cortical lesion severity or the cortical hemorrhagic load suggesting a separate pathology. Instead, the results indicate choroidal injury as one driver of the post-traumatic hydrocephalus, since the higher the choroid plexus iron load the larger were the ventricles at 6 months. The ventricle size or the choroid plexus iron load did not associate with seizure susceptibility. Cortical hypoperfusion and memory deficits were worse in rats with greater ventricular growth.

**Supplementary Information:**

The online version contains supplementary material available at 10.1007/s00429-021-02395-5.

## Introduction

Progressive ventricular enlargement is one of the radiological hallmark features after traumatic brain injury (TBI) both in patients and in animal models (Brezova et al. 2014; Kowalski et al. 2018; Marmarou et al. 1996). The chronic ventricular enlargement has been linked with worse outcome in TBI patients (Mazzini et al. 2003; Weintraub et al. 2017; Daou et al. 2016). Post-acute, slowly progressive ventricular enlargement secondary to TBI can be driven by cerebrospinal fluid (CSF) circulation deficits (*hydrocephalus*) or atrophy (*ventriculomegaly*), or both. We study the causes and consequences of the chronic post-traumatic ventricular enlargement after experimental TBI in rat.

Causes for post-traumatic hydrocephalus are not completely understood but it can result from abnormal cerebrospinal fluid (CSF) production by choroid plexus. Choroid plexus, a highly vascularized cellular plexus along the ventricle walls and floating within the ventricles, is one of the sites secreting CSF, and the blood–CSF barrier of the choroid plexus epithelium handles the transfer of blood derived compounds and cells into the CSF (Hladky and Barrand 2016; Damkier et al. 2013). Choroid plexus may respond to the brain insult by altering its protein and peptide production and release, by increased leukocyte or progenitor cell migration, and by altered CSF production and absorption (Xiang et al. 2017). Choroid plexus injury has been observed after TBI in imaging studies in humans (Hubert et al. 2019) and tissue examinations of blast injury and controlled cortical impact injury rodent models (Kaur et al. 1996; Szmydynger-Chodobska et al. 2012).

Post-traumatic hydrocephalus has been reported to be a prognostic factor for post-traumatic epilepsy (Mazzini et al. 2003), and idiopathic hydrocephalus has been associated with epilepsy development as well (Piatt and Carlson 1996). Putative mechanistic links between enlarged ventricles and tissue hyperexcitability are increased permeability of the ventricle ependymal (Olopade et al. 2012; Szmydynger-Chodobska et al. 2009; 2012; Ge et al. 2017; Ringstad, Vatnehol, and Eide 2017) and reduced interstitial waste clearance upon CSF circulation deficits (Sullan et al. 2018; Marchi et al. 2016). Lateral fluid percussion injury (FPI) rat model is a well characterized model of post-traumatic epilepsy, where by 1 year after injury 43–50% have become epileptic (I. Kharatishvili et al. 2006). Progressive ventricular enlargement in subacute phase after FPI is a common co-morbidity which has been largely overlooked in post-traumatic epileptogenesis studies. Moreover, intracranial and intracerebral hemorrhages evoked by FPI may block the arachnoid villus preventing CSF absorption, and they have been associated with worse hydrocephalus (Zhao et al. 2014; Hu et al. 2014; Xiang et al. 2017). Intracerebral hemorrhages are also a known risk factor for early and late seizures (Zhang et al. 2014; Wang et al. 2021).

Furthermore, memory disorders have been strongly linked with the brain waste clearance deficits (Boespflug and Iliff 2018), ventricular enlargement have been suggested as one measure of Alzheimer’s disease progression (Nestor et al. 2008; Apostolova et al. 2012) and the idiopathic normal pressure hydrocephalus disease is characterized by dementia and associated with risk of Alzheimer’s (Malm et al. 2013; Luikku et al. 2019; Eide et al. 2020).

We hypothesized that the severity of the choroid plexus injury assessed by MRI would associate with the severity of the post-traumatic ventricular enlargement, and the post-traumatic ventricular enlargement to be worse in rats that become epileptic. We investigated whether the cortical hemorrhagic load would associate with the ventricle growth after FPI. Finally, we studied if the memory deficit after FPI depended on the severity of the ventricular enlargement. Taken together, our objective was to study how the severity and progression of post-traumatic ventricular enlargement is associated with (1) different drivers: choroid plexus injury or cortical hemorrhagic load, and (2) functional outcomes: memory deficit and the development of epilepsy after FPI.

## Methods

### Study design

**Fig. 1:**
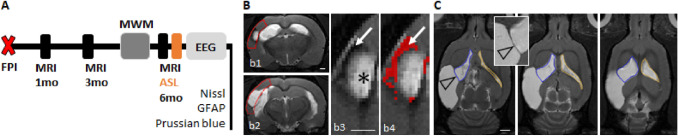
Study design*.* **A** Rats were imaged 1, 3 and 6 months after lateral fluid-percussion injury (FPI). Structural MRI was acquired at each timepoint. Blood perfusion was assessed during the 6 months scan by arterial spin labeling (ASL, described in Supplementary data). Spatial memory was tested at 5 months post-injury by the Morris water-maze (MWM). The pentylenetetrazole seizure-susceptibility test was performed at the end of 4 weeks continuous video-EEG recording. Histology included Nissl (lesion location and ependymal structure) and Prussian blue (ferritic iron) stainings. **B** Cortical lesion volume and cortical hemorrhagic load were quantified from T2-weighted (T2w) coronal images within a region of interest (ROI) matching with the shape of the intact cortex (red outline in panels b1–b2). Higher magnification images of panel b1 (panels b3 and b4) show the lesion. Both the hyperintense (asterisk in panel b3) and hemorrhagic (arrow in panels b3 and b4) portions were measured and the sum was considered to represent the cortical lesion. Pixels identified to present the hemorrhagic component of the cortical lesion, based on the prior thresholding analysis, are shown in red in panel b4. **C** Ipsilateral (blue outline) and contralateral (orange outline) ventricle volumes were manually outlined in horizontal T2w images. The insert shows the membrane separating the CSF cavity related to the cortical lesion from the ipsilateral ventricle (arrowhead). Scale bars equal 1 mm in panels b1 and b3, 2 mm in panel C

Study design of the 6-month post-TBI MRI follow-up is summarized in Fig. 1A. Imaging was performed at 1, 3, and 6 months after TBI. Morris water-maze test was conducted at 5-month post-injury. After the last MRI, electrodes were implanted for a 4-week continuous video-EEG monitoring to detect unprovoked seizures. In the end of the video-EEG, animals underwent the pentylenetetrazol (PTZ) seizure-susceptibility test before perfused for histology.

### Animals

Adult (weight 350–420 g) male Sprague–Dawley rats (*n* = 39; Harlan Netherlands B.V., Horst, The Netherlands) were used. Animals were housed in a controlled environment (temperature 22 ± 1 °C, humidity 50–60%, and light–dark cycle from 07.00 to 19.00 h) with free access to food and water. All animal procedures were approved by the Animal Ethics Committee of the Provincial Government of Southern Finland, and performed in accordance with the guidelines of the European Community Council Directives 2010/63/EU.

### Induction of TBI

Lateral fluid-percussion brain injury (FPI) was induced as previously described (I. Kharatishvili et al. 2006). Briefly, rats were anesthetized by intraperitoneal injection (6 ml/kg) of a mixture of sodium pentobarbital (58 mg/kg), magnesium sulfate (127.2 mg/kg), propylene glycol (42.8%), and absolute ethanol (11.6%). A craniectomy of 5 mm diameter was drilled between bregma and lambda with a hand-held trephine on the left convexity. Anterior edge of the craniectomy was 2.0 mm posterior to the bregma and lateral edge adjacent to the left lateral ridge. The bone was removed leaving the dura intact. Fluid-mediated impact was induced by transient pressure pulse of 21–23 ms with a fluid percussion device (AmScien Instruments, Richmond, Virginia, USA) against exposed dura. The pressure delivered ranged from 3.1 to 3.4 atm, corresponding to severe TBI. Sham-operated experimental controls underwent identical surgical procedure without impact.

### Structural magnetic resonance imaging (MRI)

MRI scanning was performed using 9.4 T/31 cm magnet (Bruker, ParaVision 5.1 system) with linear volume transmitter and quadrature surface receiver coils. During the scans animals were anesthetized with isoflurane (1.5%) with 70% N_2_O/30% O_2_ as a carrier gas, and secured to an animal holder with ear bars and bite bar. The body temperature was maintained at 37 °C throughout scanning. Respiratory rate was maintained between 60 and 70 per minute by adjusting isoflurane anesthesia level.

Structural MRI in each timepoint included T2 weighted (T2w) multi-slice fast spin echo images in coronal and horizontal orientation, and T1/T2*-weighted mixed contrast 3D gradient echo images with FID refocusing. T2w images were acquired with TurboRARE sequence with repetition time 2500 ms, effective echo time 33 ms (echo train length 8 echoes, shortest echo time 10.7 ms, echo spacing 11 ms), field of view 30 × 30 mm^2^, 256 × 256 data matrix, 6 averages and scan time 8 min. Slice set was composed of 17 consecutive coronal slices or 14 horizontal slices with thickness of 0.7 mm with no gaps. Anatomical 3D FISP images with T1/T2* mixed contrast sensitive to iron were acquired with fast imaging with steady state precession (3D FISP) sequence with repetition time 8 ms, echo time 4 ms, flip 15°, 50 kHz spectral width and 3 averages. Isotropic 175 µm^3^ resolution was obtained by FOV 40 × 40 × 40 mm and 228 × 228 × 228 matrix.

### Quantification of choroid plexus injury, cortical lesion volume and lateral ventricular volume

Severity of injury to the choroid plexus was quantified in 3D FISP images as the amount of choroid plexus iron. That is, the number of voxels below the experimentally determined intensity threshold for iron (defined below). Analysis was limited to the choroid plexus only, thereby excluding any other iron deposits. Iron stood out clearly from the background and matched closely to the iron in Prussian blue-stained histological preparations (as shown in results). The number of false positive iron pixels in automated analysis, identified by an expert visual analyzer (RI), was in worst case 6 pixels (0.03 mm^3^), and that was determined as the lower detection limit of the method.

Cortical lesion volume was determined as pixels within cortical ROI that deviated > 4 standard deviations (SDs) from the contralateral gray matter signal intensity. ROI was manually drawn, followed the shape of the intact cortex (Fig. 1B b1–b2), included all cortical T2w abnormalities as well as > 1 mm width of the surrounding normal appearing cortex dorsally and ventrally. Cortical hemorrhagic load, that is the hemorrhagic lesion volume, was the area of hypointense pixels > 5SDs below the contralateral gray matter signal intensity. Thresholds were experimentally determined to match the manually outlining of an expert reviewer (RI).

Ipsilateral and contralateral ventricle volumes were manually outlined in T2w horizontal images (Fig. 1C). Narrow membrane separating the ipsilateral ventricle from the adjacent cortical lesion volume was visible in horizontal images, and the outline of the ipsilateral ventricle followed that membrane (Fig. 1C). Consequently, the CSF volume of the cortical lesion was not included into the ipsilateral ventricle volume (and vice versa). It should be noted that the ipsilateral ventricle enlargement was confounded by the injury-induced atrophy in the ipsilateral subcortical structures, such as the thalamus and striatum. Contralateral ventricle enlargement was considered to reflect hydrocephalus due to the putative CSF circulation deficit.

To differentiate between the obstructive and non-obstructive conditions both the 3D FISP and T2w images were screened (by RI) for any blockages of the CSF circulation in foramina of Monroe, aqueduct of Sylvius, IV ventricle and sinus sagittalis.

### Morris water maze (MWM)

Spatial learning and memory was tested as a functional outcome measure at 5 months after injury, using a 3-day Morris water-maze test (Morris 1984). In MWM a black circular tank filled with water (diameter 150 cm, height 60 cm, water temperature 19 ± 1 °C) was divided into four quadrants and a platform (10 × 10 cm) was placed 1.5 cm below water level in middle of southwest quadrant of the tank. Automated video-tracking of the animal swimming pattern was done by a video camera positioned over the tank (analysis software EthoVision^®^ by Noldus, Wageningen, The Netherlands). A series of 5 trials was performed on 3 consecutive days. In each trial the animal would swim and search the submerged platform to learn the location of the platform based on visual cues. Starting point (North, South, East and West) was random for each trail. Swim time allowed was 60 s. If rat was unable to find submerged platform, it was guided to find to platform by experimenter. On day 3, an additional probe trial was conducted, where platform was removed and animal was allowed to swim for 60 s. Latency to find the hidden platform, time spent in correct quadrant in probe trial, and swimming speed were measured.

### Electrode implantation and electroencephalography (EEG)

**Fig. 2:**
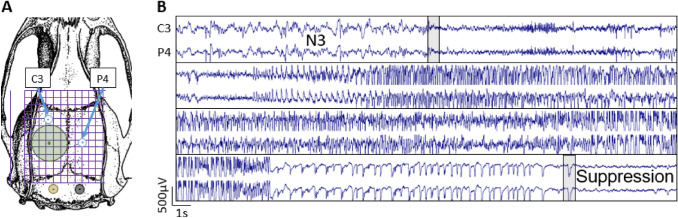
Electroencephalography recordings. **A** Location of the craniectomy (Green circle 5 mm) and electrodes [two recording electrodes (C3 and P4), ground, and reference]. **B** Representative example of a generalized seizure that started in the transition between N3 and REM sleep. Beginning and the end of the seizure are indicated by the grey bars

After the last MRI scans at 6 month post-TBI, rats underwent electrode implantation surgery. Briefly, rats were anesthetized with an i.p. injection (6 ml/kg) of sodium pentobarbital (58 mg/kg), magnesium sulphate (127.2 mg/kg), propylene glycol (42.8%) and absolute ethanol (11.6%) and mounted in a stereotactic frame. Two cortical recording screw electrodes (1 mm diameter, Plastics One Inc., Roanoke, VA, USA) were implanted as shown in Fig. 2A: one rostral to the craniectomy, and the other contralateral to the centre of the craniectomy. Two screw electrodes serving as reference and ground electrodes were inserted into the skull bilaterally over the cerebellum. All electrode pins were inserted into the plastic pedestal, which was then cemented to the calvarium with dental acrylic.

To detect spontaneous epileptic seizures, a 4 weeks continuous video-EEG recording started 6–7 days after electrode implantation. EEG was recorded using Nervus EEG recording system with Nervus magnus 32/8 amplifier (Taugagreining, Iceland) with a sampling rate 2048 Hz. Signal was high-pass filtered with 0.3 Hz cutoff and low-pass filtered with 100 Hz cutoff. Video camera positioned above the cages was used to record the animal behavior. The video-EEG was recorded 24/7 for 4 weeks. The EEG (Fig. 2B) was analyzed first utilizing an in-house developed algorithm in Spike2 (ver.9; for details see Andrade et al 2018). After the automatic detection the results (including false positives) were verified visually using Spike2 (TP, PA).

### Pentylenetetrazol seizure-susceptibility test

On the last day of the video-EEG rats were subjected to the pentyleneterazol (PTZ) chemoconvulsant challenge to assess if the animals had a lowered seizure threshold. Briefly, PTZ (25 mg/kg, 18 mg/ml dissolved in 0.9% NaCl) was injected intraperitoneally and the rat was returned back to its individual plexi-glass cage for monitoring. EEG activity and animal behaviour were assessed for the following 2 h. Latency to the first spike, number of spikes during the first 60 min and the number of induced seizures during the first 60 min after PTZ administration were analyzed.

### Tissue fixation and histochemistry

After finishing the PTZ-test (7 months post-injury or sham-operation), rats were perfused transcardially for histology. Intracardial perfusion, 4% paraformaldehyde fixation and post-fixation protocols have been described previously (Irina Kharatishvili et al. 2007). After post-fixation, brains were cryoprotected in 20% glycerol in 0.02 M potassium phosphate buffer (pH 7.4) for 48 h, frozen in dry ice and stored at − 70 ºC. Brains were sectioned (1-in-10 series, 25 µm thick, coronal plane) on a sliding microtome. First series of sections was collected in 10% formalin at room temperature and remaining series were stored in tissue collection solution (30% ethylene glycol, 25% glycerol in 0.05 M sodium phosphate buffer) at − 20 °C until further processing.

The first series of sections was stained for thionin (**Nissl**) to assess the brain cytoarchitecture and lesion location. An adjacent series of sections was stained for **Perls’ Prussian blue** to identify ferric (Fe^3+^) iron as described previously (Liu et al. 2014). Briefly, sections were incubated in freshly prepared solution of 5% potassium hexacyanoferratetrihydrate and 5% hydrochloric acid for 30 min and then rinsed with water and counterstained with nuclear fast red. The sections were then mounted on gelatin-coated slides and covered with DePex^®^. Cytoarchitectonic location and distribution of iron residues were compared to that in MRI.

### Statistics

Statistical analysis was performed using IBM SPSS Statistics 23.0 for Windows (SPSS Inc., IL, USA). Bivariate correlations were assessed by Pearson correlation coefficient with Bonferroni correction for multiple comparisons. Group differences were determined by Student’s *t* test for the volumes derived from MRI data (normally distributed), and Mann–Whitney *U* test for EEG and Morris water maze data (not normally distributed). Data are shown as mean ± standard deviation (std). *p* value < 0.05 was considered significant.

## Results

### Mortality, occurrence and duration of post-impact seizure-like behaviors, and apnea time

#### Acute post-impact mortality (< 48 h)

Acute post-impact mortality was 36% (11/31). Follow-up mortality was 0%

#### Post-impact seizure-like behavior

Acute post-impact seizure-like behavior was observed in 48% (15/31) rats. Mean duration of the jerks was 30 ± 9 s.

#### Apnea

Apnea duration after the impact was 31 ± 17 s with two rats having apnea duration > 50 s.

### Choroid plexus accumulates iron in 90% of rats with severe TBI which can be monitored in vivo with 3D high-resolution FISP MRI

All rats with TBI developed unilateral cortical lesion with variable degree of intracortical hemorrhages. They also showed signs of diffuse axonal injury-related microbleeds along the white matter tracts, particularly in the corpus callosum and external capsule. Unexpectedly, the visual survey of high-resolution 3D FISP images revealed a distinct negative contrast in the choroid plexus of the TBI rats, suggesting iron accumulation due to post-impact bleeding.

**Fig. 3:**
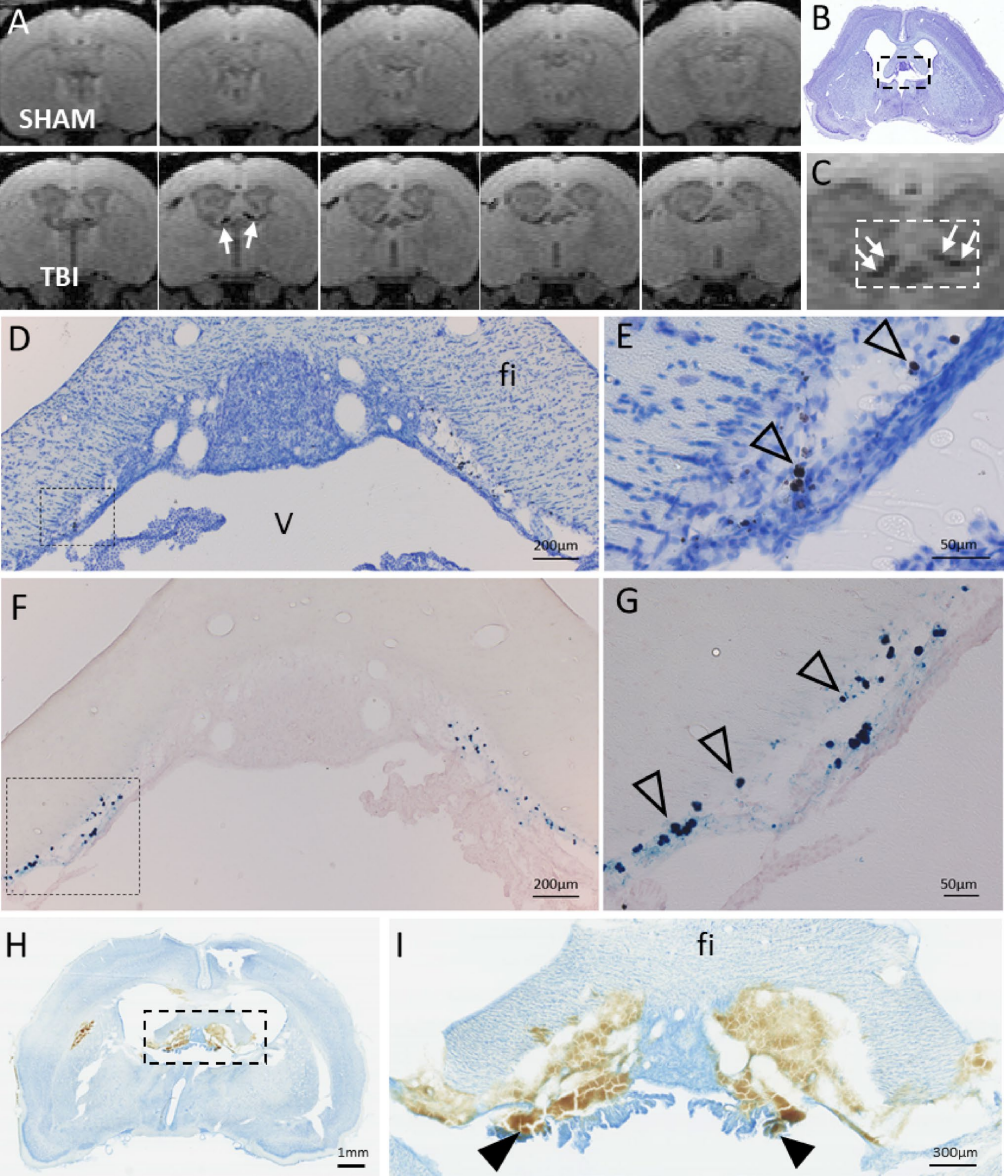
T1/T2* mixed-contrast FISP MRI detects post-traumatic iron in the choroid plexus. **A** Five consecutive 175 µm thick coronal MRI slices (centered at − 1.00 mm from bregma) of the sham-operated (top row) and TBI animal (bottom row) at 6 months post-operation. Note black signal-void areas (white arrows) in the TBI rat which are related to the presence of iron deposits in the choroid plexus. **B** Thionin-stained section of the same animal sectioned at the corresponding coronal level (7 months post-TBI). **C** Magnification of the MRI showing the choroidal iron (white arrows). Dashed box equals to that in B, and the corresponding area is shown in photomicrographs taken from thionin **D** and Prussian blue **F** stained sections. Iron deposits (arrowheads) are robustly detected along the ependymal lining at ventricular wall adjacent to fimbria in **D**, **E** thionin and **F**, **G** adjacent Prussian blue preparations. Iron appears as both diffuse and intracellular deposits. Note the match in location of iron deposits in histologic sections and MRI. **H** Coronal thionin-stained section showing acute post-impact hemorrhages in the choroid plexus and fimbria at 6 h post-TBI (sections were available from the EPITARGET tissue bank; (Lapinlampi et al. 2017). **I** Higher magnification photomicrograph of the area indicated with a dashed box in panel H. Note detachment of the ependymal layer along the ventral aspect of the fimbria (fi). The bleeds extend to the “floating part” of the choroid plexus (arrowheads). Abbreviations: cp, choroid plexus; fi, fimbria of hippocampus; V, 3rd ventricle

Our further analysis indicated iron deposits in parts of the choroid plexus, floating in the lateral ventricles as well as along the ependymal lining of the fimbria (Fig. 3). Iron deposits were present in 90% (18/20) of the injured rats at 1 month post-TBI and remained visible even 6 months post-injury. The MRI-quantified volume of iron deposits ranged from 0.04 to 0.59 mm^3^ at 1–6-month post-injury (see below). End-point histopathology using thionin and Prussian blue stainings confirmed iron accumulation in the stroma of the choroid plexus, matching with the FISP MRI finding (Fig. 3A–G). The two (2/20) TBI animals without visible iron deposits in FISP images had iron volumes below the detection limit (≤ 0.03 mm^3^). None of the sham animals had choroid plexus iron in MRI or histopathology.

To investigate the temporal evolution of iron deposits in the choroid plexus, we examined thionin-stained preparations sampled at different post-TBI timepoints, which were available in our FP7-EPITARGET tissue bank (Lapinlampi et al. 2017; Andrade et al. 2018). The analysis revealed acute bleeds in the choroid plexus and fimbria already at 6 h post-TBI, which matched with the location of iron deposits in FISP MRI at 1–6 months post-TBI (Figs. 2H–I, 3).

### Progressive chronic ventricular enlargement in rats with TBI

#### Lateral ventricle volume

In sham-operated experimental controls (*n* = 8) the ventricular volume 3 months after operation was 3.8 ± 2.3 mm^3^ (range 3.1–5.1 mm^3^) ipsilaterally and 3.3 ± 0.4 mm^3^ (range 2.8–4.0 mm^3^) contralaterally (ipsilateral vs. contralateral *p* > 0.05, paired *t* test). At 6 months, the volumes were 4.1 ± 0.8 mm^3^ ipsilaterally and 3.5 ± 0.4 mm^3^ contralaterally (ipsilateral vs. contralateral *p* > 0.05) displaying minor but significant bilateral growth (*p* < 0.05 as compared to ventricular volumes at 3 months).

**Fig. 4:**
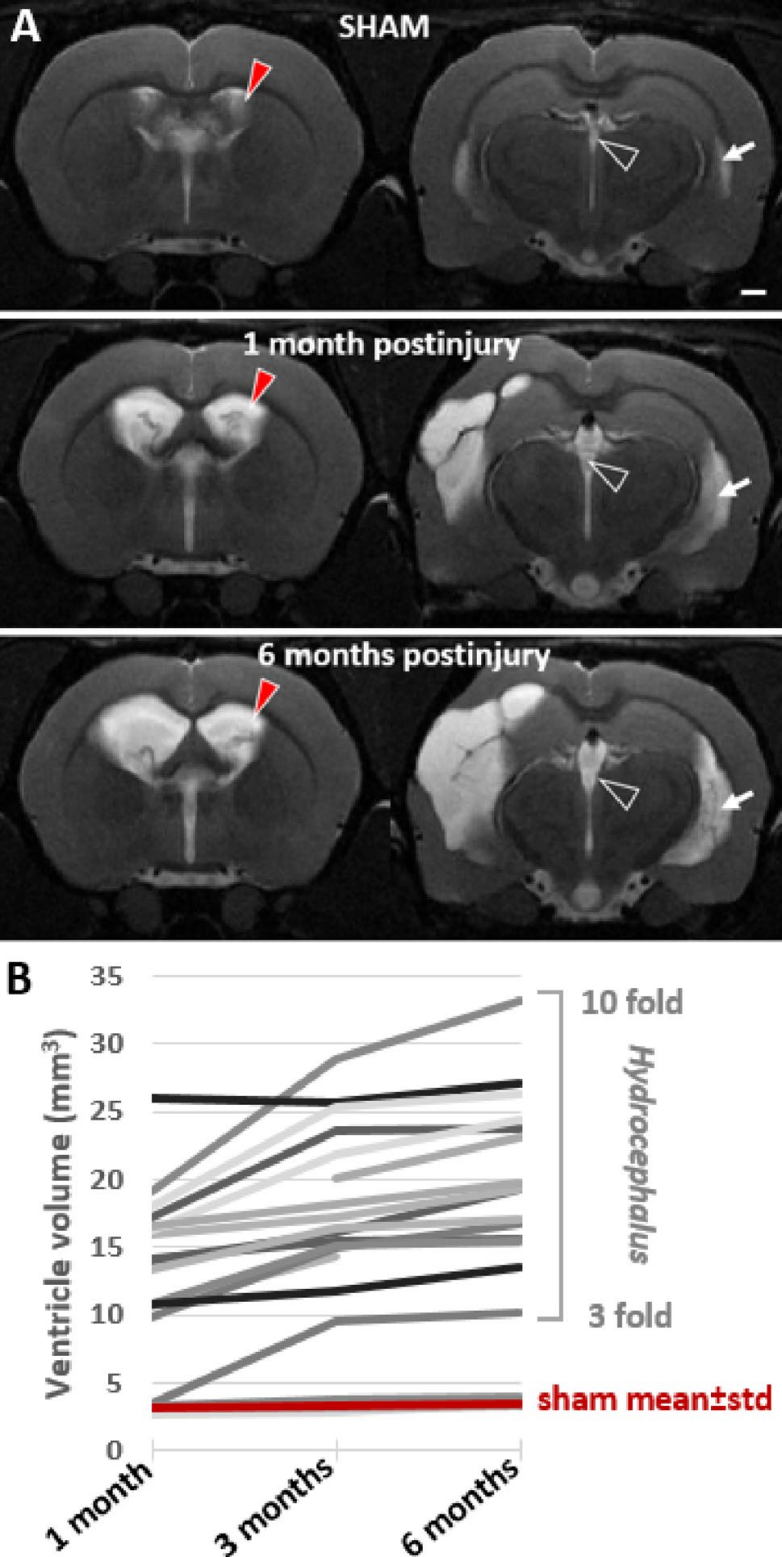
Ventricular enlargement continued for 6 months after lateral fluid-percussion injury. **A** T2-weighted image pair shows ventricular enlargement at rostral (− 0.9 mm from bregma) and caudal (− 3.8 mm from bregma) levels. Cerebrospinal fluid is seen as a bright signal (arrow). Top row: a representative sham-operated experimental control. Mid row: a rat with TBI imaged at 1-month post-injury. Note that a unilateral impact force induced a bilateral ventricular enlargement. Bottom row: the same rat imaged at 6-month post-injury (contralateral ventricle volume > 25 mm^3^). Contralateral ventricle volume (red arrowhead) was used as a measure of post-traumatic hydrocephalus severity. **B** Threefold-to-tenfold enlargement of the contralateral ventricle occurred in 17/20 rats with TBI. Only 3/20 rats with lateral FPI had ventricle volumes corresponding to that in sham-operated experimental controls (3.1 ± 0.4 mm^3^ at 1 month, 3.3 ± 0.4 mm^3^ at 3 months, 3.5 ± 0.4 mm^3^ at 6 months). Ventricle enlargement was observed also in the lateral horn of the lateral ventricle (white arrow) and in the 3rd ventricle (open arrowhead)

In rats with TBI, the ventricular volumes expanded over time bilaterally, even though the progression was more pronounced in the injured hemisphere (Fig. 4A). At 3 months post-TBI, ventricular volumes were 27.2 ± 8.0 mm^3^ ipsilaterally (*p* < 0.001 as compared to the sham-operated experimental controls) and 16.4 ± 7.5 mm^3^ contralaterally (*p* < 0.001, *n* = 20). At 6 months post-TBI, ventricular volumes had enlarged to 34.7 ± 11.9 mm^3^ ipsilaterally (*p* < 0.001 as compared to 3 months) and 17.5 ± 8.4 mm^3^ contralaterally (*p* < 0.001, *n* = 18). The ventricular volumes continued to grow between the 3 and 6 months in 50% of rats (9/18) contralaterally and in 15/18 (83%) ipsilaterally. Figure 4B shows the contralateral ventricle growth over time in individual animals.

Lateral ventricle ratio (LVR) was calculated as the ratio of ipsilateral to contralateral lateral ventricle volume. In sham-operated controls the ratio was 1.14 ± 0.19 at 3 months and 1.15 ± 0.20 at 6 months (*p* > 0.05).

In rats with TBI, the ratio was 2.17 ± 1.72 at 3 months and 2.58 ± 2.17 at 6-month post-injury (*p* < 0.001). Only one of the rats with TBI had a unilaterally dominant ventricular enlargement between the 3 and 6 months, the LVR growing from 8.89 to 10.85. Other injured animals showed a bilateral, even though ipsilaterally dominant ventricular enlargement. There was a correlation between the ipsilateral and contralateral ventricle enlargements (*r* = 0.721, *p* < 0.0001, *n* = 20 at 3-month post-TBI).

### Mechanical obstructions of the CSF circulation were not found

Next we screened both the 3D FISP and T2w images for the presence of any mechanical obstruction that could explain the ventricle enlargements, including (i) foramen of Monro, (ii) aqueduct, and (iii) 3rd or 4th ventricle. No obstructions were found that would block the CSF flow. Our analysis did not allow a complete exclusion of obstruction in the sagittal sinus, because the FISP contrast was not optimized for that and in T2w images the pulsation artefact in phase direction masks the sagittal sinus (cross section in coronal view) partially. Even though the choroid plexus iron was detected bilaterally along the interventricular foramina connecting the lateral ventricles to the 3rd ventricle, no mechanical obstruction between right and left ventricles were observed. Rather, we found an expansion of the connecting canals and of the 3rd ventricle. Hence, the CSF flow in the ventricular system does not appear to be limited.

### Choroid plexus iron load reaches maximum by 1 month, remains stable thereafter, and associates with the ventricle enlargement after TBI

**Fig. 5:**
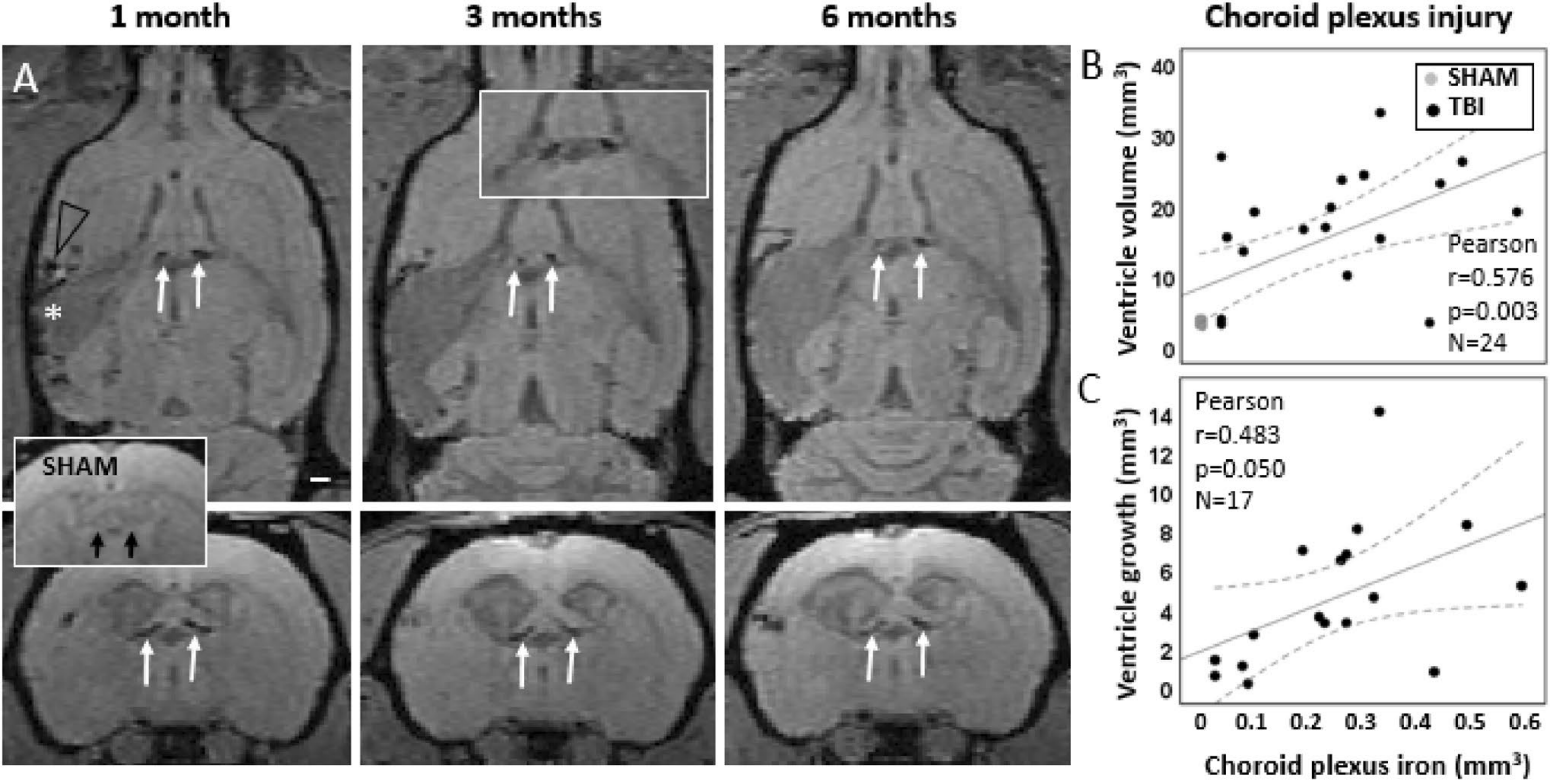
In vivo follow-up of choroid plexus injury and its association with ventricle enlargement. **A** MRI follow-up with T1/T2* mixed contrast FISP reveals that iron accumulation in the choroid plexus is bilateral (pairs of white arrows) and stable from 1- to 6-month post-injury. Sham-operated experimental controls had no iron in the choroid plexus (insert with black arrows). In the lesioned cerebral cortex, iron (black arrow head) surrounds the lesion cavity (white asterisk). **B** Chronic choroid plexus iron load at 6 months post-TBI correlated with the contralateral ventricle volume at 6 months (*p* < 0.01). **C** Greater the choroid plexus iron load at 1 month, the greater the subsequent contralateral ventricular growth from 1 to 6 months (*p* ≤ 0.05). Pearson correlation is displayed with linear regression line with 95% confidence intervals

To study the role of the choroid plexus injury as a driver of the ventricular enlargement, we next investigated the amount of iron in the choroid plexus over time (Fig. 5**)**.

Sham-operated experimental controls had no iron in the choroid plexus (volume was < 0.011mm^3^).

At 1-month post-TBI, the quantified choroid plexus iron load was 0.27 ± 0.17 mm^3^ (range 0.03–0.59 mm^3^), at 3 months 0.26 ± 0.16 mm^3^ (range 0.02–0.57 mm^3^) and at 6 months 0.24 ± 0.16 mm^3^ (range 0.04–0.58 mm^3^). There was no progression in choroid plexus iron load in rats with TBI as the change in the amount of iron between the 1- and 6-month timepoints was only 0.009 ± 0.031 mm^3^. The largest individual increase in iron amount was 0.032 mm^3^, which barely exceeds the detection limit of 0.03 mm^3^. The largest individual decrease in iron load over 1–6 months was 0.16 mm^3^, but in all other cases the decrease was ≤ 0.038 mm3. The mean standard deviation of the 3 measurements (1, 3 and 6 months) of iron load in individual animals was 0.018 mm^3^, which is below the 0.03 mm^3^ detection limit. Thus, from 1- to 6 months post-TBI there was no further choroidal iron accumulation observed, and indication of iron clearance was detected only in one rat.

Correlation analysis between the choroid plexus iron load and ventricular enlargement indicated that the greater the choroid plexus iron load, the larger the contralateral ventricle volume (at 1 month *r* = 0.466, *p* < 0.05, *n* = 24; at 3 months *r* = 0.596, *p* < 0.001, *n* = 27; at 6 months *r* = 0.576; *p* < 0.01, *n* = 24, Pearson) (Fig. 5B). Examining the interrelation among only the injured rats yielded a similar non-significant trend. Moreover, the greater the choroid plexus iron load at 1 month, the greater the subsequent contralateral ventricular growth from 1 to 6 months (TBIs and shams combined *r* = 0.651, *p* < 0.001; rats with TBI only *r* = 0.483, *p* = 0.05, Pearson) (Fig. 5C).

### Progression of the cortical lesion atrophy and cortical hemorrhagic load did not associate with ventricular enlargement

#### Cortical atrophy

Sham-operated experimental controls had no cortical lesion. All rats with TBI developed a cortical lesion. Cortical lesion volume was assessed as a combined volume of the hemorrhagic lesion and the hyperintense lesion, and the final volume at 6 months post-injury ranged from 10.6 to 86.3 mm^3^ At 1 month post-injury the lesion volume was 37.66 ± 24.06 mm^3^, at 3 months 43.99 ± 24.13 mm^3^ and at 6 months 46.64 ± 23.77 mm^3^. Due to the great variation in lesion volumes there was no group difference between the timepoints (*p* > 0.05). However, in individual TBI rats the increase of the lesion volume from 1 to 6 months ranged from 1.40 to 22.78 mm^3^ which corresponded to 1.7% to 141.1% increase in lesion volume. The lesion growth greater than 1.5 mm^3^ (detection limit) was observed in 75% (15/20) TBI rats (for details see Yasmin et al., 2019).

#### Volume of cortical hemorrhagic lesion

Hemorrhagic lesion volume in T2w images (see Fig. 1B) was used as a surrogate marker for cortical bleeding. Sham-operated experimental controls did not show any signs of cortical hemorrhage.

In rats with TBI, the volume of the hemorrhagic cortical lesion ranged from 0.23 to 9.13 mm^3^ at 1 month post-injury, and from 0.30 to 8.07 mm^3^ at 6 months post-injury. At 1 month post-injury the hemorrhagic lesion volume was 3.25 ± 2.11 mm^3^, at 3 months 2.21 ± 1.96 mm^3^ and at 6 months 1.98 ± 1.86 mm^3^. Thus, unlike in the choroid plexus, the cortical hemorrhagic volume decreased over time (*p* < 0.01, 1 vs. 3 months and 3 vs. 6 months, Wilcoxon) as the hemorrhagic regions atrophied and merged into a cortical lesion cavity.

#### Association with ventricular enlargement

Neither the ipsilateral nor the contralateral ventricle volume correlated with cortical lesion volume at 3 or 6 months post-injury (*p* > 0.05). Ventricle growth between 3 and 6 months also did not correlate with corresponding growth of the cortical lesion (*p* > 0.05). That is, massive ventricular enlargement was also seen in rats with small focal cortical lesions. This suggests that the mechanisms driving the chronic ventricle expansion are independent of those driving the chronic atrophy in the injured cortex.

The volume of the cortical hemorrhagic lesion did not correlate with the contralateral ventricle volume or ventricle growth (*p* > 0.05).

### Choroid plexus iron load have no association with seizure susceptibility

Since iron related to cortical and intraventricular bleeds can induce acute seizure activity and even associate with the later development of post-traumatic epilepsy (Willmore and Rubin 1981; Sharma et al. 2007; Dadas and Janigro 2019) we next investigated the association of cortical and choroid plexus iron with the seizure susceptibility using pentylenetetrazol (PTZ) chemoconvulsant challenge.

At 6-month post-TBI, 17 of 20 rats with TBI survived and underwent a 4-week continuous vEEG monitoring. Only 1 rat with TBI (case #10) showed spontaneous unprovoked seizures in vEEG. This rat had small choroidal iron load of 0.08 mm^3^ at 1 month post-injury.

The PTZ test performed in the end of vEEG was successful in 16 of 17 TBI animals (PTZ injection failed in one TBI and in one sham-operated rat). Latency to the first spike after PTZ injection was 213 ± 77 s in sham operated rats and 169 ± 59 s in TBIs (*p* > 0.05). Four of 16 (25%) TBIs had latency shorter than 137 s (sham mean minus one standard deviation) indicating elevated seizure susceptibility. No group difference was found in number of PTZ-induced seizures, latency to the first seizure, or seizure duration. Three of 6 shams (50%) had one seizure after PTZ. Eight of 16 TBI rats (50%) had 1 seizure, and one 2 seizures. The mean latency to the seizure was 1027 ± 616 s in shams and 1267 ± 1751s in rats with TBI (*p* > 0.05). The mean duration of induced seizures was 103 ± 48 s in shams and 116 ± 98 s in rats with TBI (*p* > 0.05).

**Fig. 6:**
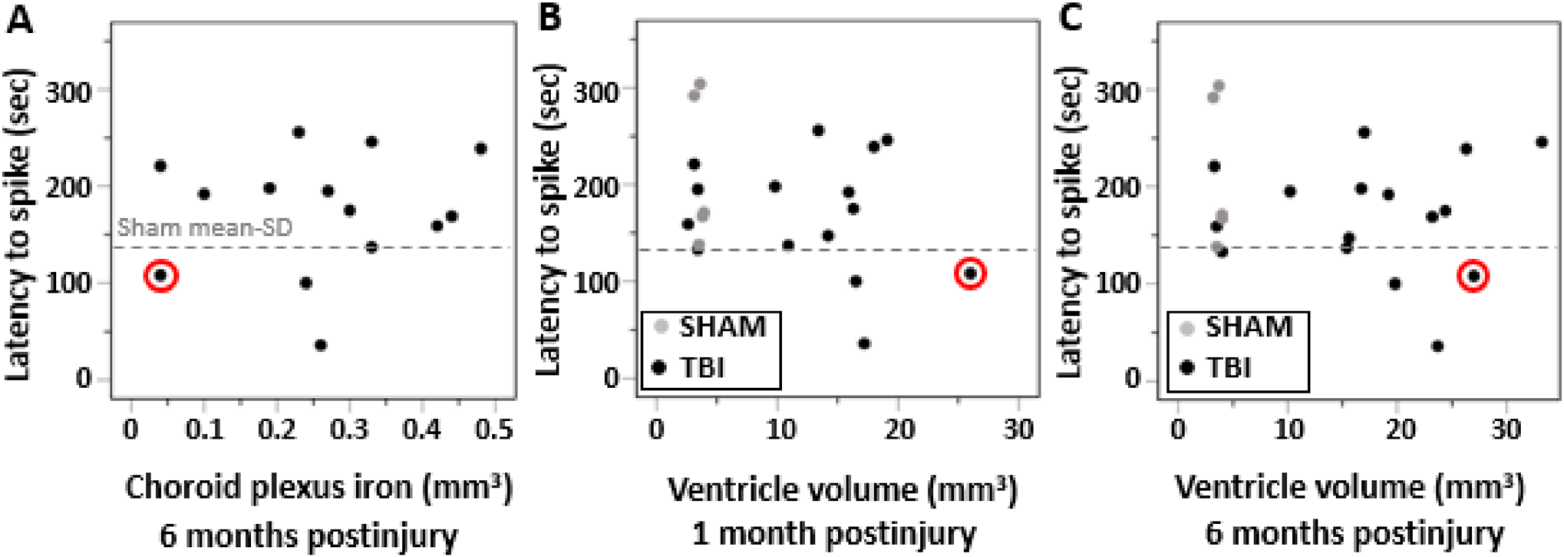
Choroid plexus iron load or the contralateral ventricle enlargement did not associate with seizure-susceptibility in the PTZ test. **A** Volume of choroid plexus iron at 6 months, **B** ventricular enlargement at 1 month or **C** at 6 months did not associate with seizure susceptibility in the pentylenetetrazole (PTZ) test (*p* > 0.05, Pearson). Note that the rat that developed spontaneous seizures (case # 10, circled in red) was the one with the largest contralateral ventricle enlargement at 1 month

At 1 month post-TBI, choroid plexus iron was found in 14 out of 16 TBI rats undergoing PTZ test 6 months later. The iron was still present at 6 months in all of these animals. The amount of choroid plexus iron at 6 months did not correlate with latency to first spike in PTZ test at 7 month post-injury (*r* = 0.111, *p* = 0.705, *n* = 14, Pearson) (Fig. 6A). The volume of the choroid plexus iron in the 4 TBI animals with increased seizure susceptibility did not differ from that in the remaining TBI animals. This suggests that choroid plexus iron load is not linked with tissue hyperexcitability.

Since the thinner ependymal and glial lining related to severely expanded ventricles can result dysregulated infiltration of pro-epileptogenic molecules and cells into the parenchyma (Olopade et al. 2012; Szmydynger-Chodobska et al. 2009, 2012; Ge et al. 2017), we also investigated if the ventricle size associated with seizure susceptibility. Even though the rat with spontaneous seizures (case #10) had the largest contralateral ventricle volume at 1 month (26 mm^3^) and the second largest contralateral ventricle at 6 months (27 mm^3^), we did not find any correlations between the ipsilateral and contralateral ventricle volumes either at 1 month or 6 months and the latency to first spike in the PTZ-test (Fig. 6B, C).

### The more severe the hydrocephalus the poorer the spatial memory

**Fig. 7:**
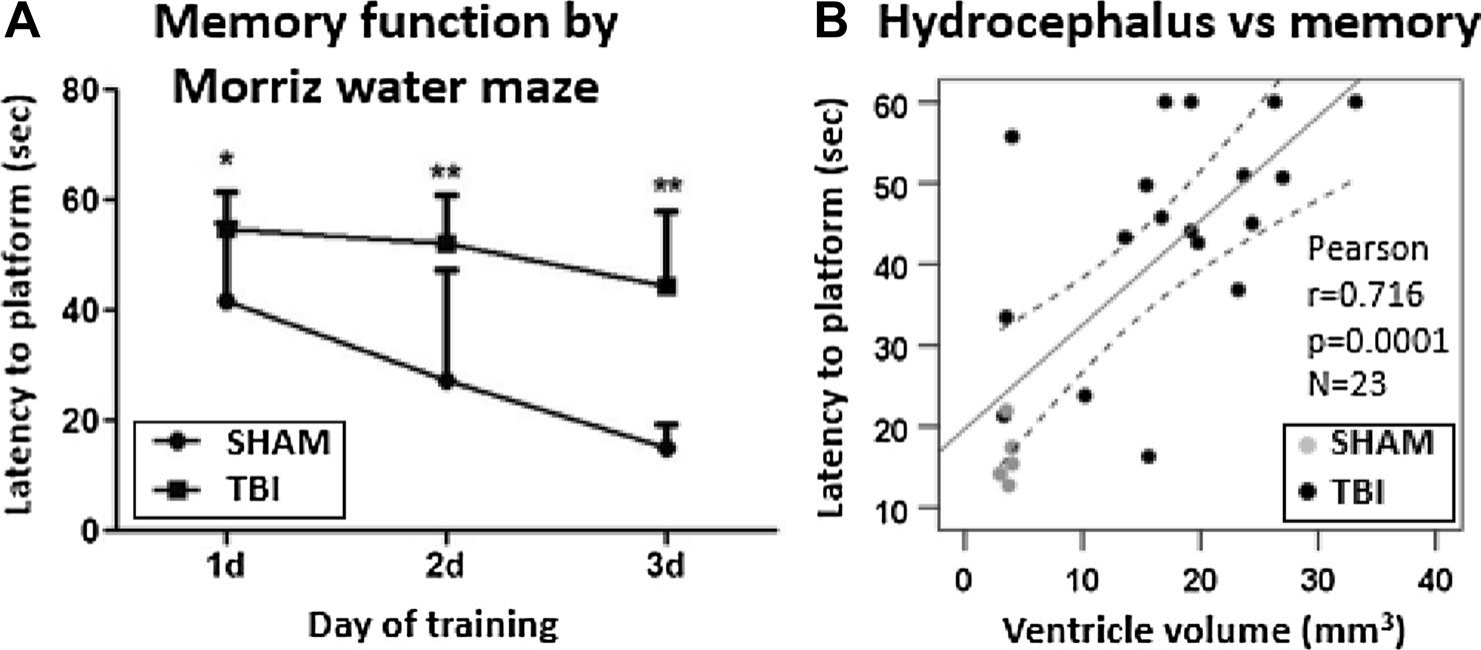
Spatial learning and memory performance in the Morris water-maze test*.* **A** Latency to find the submerged platform was longer in rats with lateral fluid-percussion injury as compared to sham-operated experimental controls on all testing days. **B** Latency to the platform on the last trial of the 3rd training day was used as a measure of memory performance. We found that the larger the volume of the contralateral lateral ventricle, the longer the latency to the platform at 6 months post-injury (all animals *r* = 0.716, *p* < 0.001, n = 23; TBI animals only *r* = 0.505, *p* < 0.05, *n* = 18). Statistical significances: *p < 0.05, **p < 0.01 (Mann–Whitney at each timepoint corrected for multiple comparisons). Pearson correlation is displayed with linear regression line with 95% confidence intervals

Post-TBI ventricular enlargement is one sign of chronic post-traumatic encephalopathy (Brezova et al. 2014; Kowalski et al. 2018; Marmarou et al. 1996) and a risk factor for later cognitive memory deficits (Mazzini et al. 2003; Apostolova et al. 2012; Weintraub et al. 2017; Daou et al. 2016; Kowalski et al. 2018). In addition, the rats with induced hydrocephalus show impairment in Morris water maze test (Chen et al. 2017; Qi et al. 2017). We performed MWM test of spatial memory at 5 months post-TBI and found impaired performance in TBI rats. Mean latency to the hidden platform was 3 times longer in the TBI group as compared to sham-operated controls (44.4 ± 13.6 vs. 15.2 ± 4.3 s, *p* < 0.001) (Fig. 7A). Rats with TBI also showed a trend towards reduced time spent in the correct quadrant during the probe trial (14.7 ± 4.1 vs. 19.1 ± 6.7 s, *p* = 0.058). Swimming speed was comparable in the TBI and sham groups (28.3 ± 1.9 vs. 27.7 ± 4.8 cm/sec, *p* = 0.786).

The greater the contralateral ventricle volume at 6 months post-injury the longer the latency to the platform in the last day of testing (*r* = 0.716, *p* < 0.001, *n* = 23). Importantly, similar correlation was found in the TBI group only (*r* = 0.505, *p* < 0.05, *n* = 18) (Fig. 7B).

## Discussion

Our objective was to investigate the evolution of hydrocephalus and its association with epileptogenesis and memory impairment after TBI. Hydrocephalus was defined as contralateral ventricular enlargement after unilateral FPI. We had 6 major findings. First, ventricular enlargement continued bilaterally for 6 months. Second, the evolution of ipsilateral or contralateral ventricular enlargement did not correlate with the cortical atrophy, turning our focus on search of other factors which could explain the hydrocephalus. Third, we found a consistent iron accumulation in the choroid plexus hanging in the ventricles rostral to the fimbria. Fourth, the choroid plexus hemorrhage was detectable already at 6 h post-impact, remaining stable thereafter. Fifth, the greater the choroid plexus iron accumulation, the more severe the hydrocephalus. These data suggest that the impact-induced choroid plexus hemorrhage, iron accumulation, and consequent CSF circulation deficit contribute to ventricle enlargement after lateral FPI-induced TBI. Sixth, the choroid plexus iron load nor the ventricular growth did not associate with seizure susceptibility. However, the more severe the hydrocephalus, the more impaired the performance in spatial memory test.

### Choroid plexus hemorrhage and iron accumulations occur rapidly after lateral FPI

Hydrocephalus or ventriculomegaly are common after human TBI (Marmarou et al. 1996; Kowalski et al. 2018) and occur with increased or normal intracranial pressure (Marmarou et al. 1996). Ventricular enlargement is also a common finding in animal models of TBI (Bramlett and Dietrich 2002; Ding et al. 2013; Turtzo et al. 2013). In the rat lateral FPI model we found a growth of ipsilateral ventricles up to 1340% (14-fold) as compared to the volume in shams, and contralateral ventricles up to 703% (eightfold) over the 6 months follow-up, which were not related to the extent off the cortical atrophy. Previous studies with measurements of intracerebral pressure (ICP) have showed only acute increases in ICP, up till 24 h post-FPI (recent review by (Glushakova et al. 2020) with no evidence of chronic increase in the ICP (to our knowledge). Brain swelling due to transient acute and subacute edema is mostly resolved by 9-day post-FPI, and FPI-craniectomy acts as ‘decompressive craniectomy’. This makes elevated ICP an unlikely etiology for the chronic progressive ventricle enlargement. Unlike expected, we did not find any association between the severity of ipsilateral cortical atrophy and ventricle enlargement. Moreover, progressive ventricle enlargement was present also contralaterally, supporting the idea that some ongoing active process such as a TBI-induced deficit in CSF secretion, absorbtion or circulation is driving the distension of ventricular system, leading to (normal pressure) hydrocephalus.

CSF is produced by choroid plexus distributed along the ventricular walls. After exposure of the epidural space to fluid-percussion impact force (2.6–2.9 atm), the pressure in the lateral ventricles shows an abrupt bilateral increase from 1.0 to 2.7–2.9 atm (Clausen and Hillered 2005). The pressure wave travelling in the closed intraventricular space can distort and rupture the highly vascularized choroid plexus and detach the ependymal layer of the ventricular walls, leading to hemorrhage and accumulation of iron. Our current histologic analysis showed massive choroid plexus hemorrhage already at 6 h after the impact. The most prominent choroid plexus injury found at the level of the foramina of Monro, which connect the lateral ventricles to the 3^rd^ ventricle, suggesting one possible location for a TBI-induced CSF circulation deficit. Moreover, the ependymal veins, aligning the fimbria-ventricle border present another structure suspect to shearing injury related to intraventricular pressure wave. In correspondence, in vivo MRI revealed choroid plexus iron accumulation as diffuse hypointensity at 2-day post-FPI which intensified by 9-day post-FPI and persisted for the 6 months follow-up (Suppl. Figure 1). However, no iron was found elsewhere in the ventricular walls, which may also relate to the resolution of MRI. We observed iron also along the internal cerebral veins that drain the choroid plexus (Suppl. Figure 2). However, only 4/18 rats with choroidal iron had iron also along internal cerebral vein suggesting that to be an independent finding rather than a downstream effect. Analysis of animals imaged in our other ongoing studies (EPITARGET www.epitarget.eu and EpiBioS4Rx www.epibios4rx.edu) revealed choroid plexus iron in most of the animals (unpublished).

We did not observe any obstructions in the ventricular canals. Rather, the ventricular system and foramina appeared dilated, being open for free non-obstructed CSF flow. Another etiology for ventricular enlargement could be clotting in the veins associated with the CSF drainage, such as the sagittal sinus. We found some abnormalities in consecutive coronal MRI slices along the sagittal sinus in a subset of injured rats. However, the finding were not conclusive as image artifacts from pulsatile blood flow of sagittal sinus itself and of internal cerebral vein propagate along the phase encoding direction, project over sagittal sinus and can mimic focal thrombosis (Stadler et al. 2007). Furthermore, recent imaging studies in rats suggest that the CSF is drained primarily via the spinal canal and olfactory route rather than via sagittal sinus (Murtha et al. 2014). One etiology for progressive ventricle enlargement could relate to the impaired function of the glymphatic system (Jessen et al. 2015; Ren et al. 2013). Perfusion deficits due to reduced blood flow, low arterial pulsation pressure and vascular deficits can hinder the fluid transport along the perivascular space and reduce interstitial clearance (Kress et al. 2014). We found that low cortical perfusion indeed correlated with the severity of hydrocephalus (Suppl. Figure 3), suggesting that reduced CSF flow through the cortical parenchyma may be an additional mechanism contributing to the chronic post-TBI ventricular growth.

To our best knowledge, the present study is the first description of choroid plexus iron accumulation in lateral FPI model, suggesting compromised brain–CSF barrier function. One mechanism, how this could contribute to chronic brain atrophy and tissue hyperexcitability relates exposure of brain tissue to chronic inflammation as the previous studies have shown that immune cell can enter the CSF space via choroid plexus after TBI (Szmydynger-Chodobska et al. 2012) and stroke (Ge et al. 2017). Further support to this idea was provided by MRI studies on phagocytic cells, containing superparamagnetic iron oxide contrast particles and located in choroid plexus stroma, which were found to pass through the choroid plexus after stroke and experimental autoimmune encephalomyelitis (Wiart et al. 2007; Henning et al. 2009) (Millward et al. 2013; 2019). Moreover, a pressure wave used to induce blast injury caused widening of intercellular spaced between the epithelial cells, leading to passage of monocytes/lymphocytes across the epithelium (Kaur et al. 1996).

### Ventricular enlargement or the choroidal iron did not associate with increased seizure susceptibility but ventricular enlargement correlated with poor spatial memory

Previous studies have suggested that iron detected in the MRI as a surrogate marker to TBI-related hemorrhage is a factor contributing to epileptogenesis (Sharma et al. 2007; Mishra et al. 2013; Dadas and Janigro 2019). In the present cohort, only one of the injured animals expressed spontaneous seizures during the 4-week vEEG on the 7^th^ post-TBI month. However, 25% of the rats showed increased seizure susceptibility in the PTZ test. Even though the rat with PTE had the most enlarged contralateral ventricle at 1 month post-injury, we could not find any association between the severity of the ventricle enlargement and the seizure susceptibility. Nor could we associate the iron load in the choroid plexus with the seizure susceptibility. This suggest that at the chronic stage post-injury the choroidal iron residues are not evoking any acute stress to the CNS or promoting tissue hyperexcitability, but have been prohibited by being phagocyted, encapsulated or packed in an inert form.

Interestingly, we found that the severity of ventricle enlargement correlated with impairment in spatial memory. The progressive hippocampal atrophy occurring in parallel with the ventricle expansion also contributes to memory impairment. Lateral FPI model could be a useful tool for mechanistic analyses of hydrocephalus-related memory impairment as post-traumatic hydrocephalus associates with worsened functional outcomes and longer duration of post-traumatic amnesia in patients with TBI (Kowalski et al. 2018).

## Conclusions

We provided evidence that structural MRI provides a useful in vivo tool for analysis of choroid plexus injury after TBI and its association with functional impairments. Whether the choroid plexus iron is a surrogate marker for chronic inflammatory response, leading to ventricle enlargement and functional deficits such as memory impairment remains to be further explored.

## Supplementary Information

Below is the link to the electronic supplementary material.

Supplementary file1 (PDF 866 KB)

## Data Availability

The data sets generated during and/or analysed during the current study can be made available from the corresponding author on reasonable request.

## References

[CR1] Andrade P, Paananen T, Ciszek R, Lapinlampi N, Pitkänen A (2018). Algorithm for automatic detection of spontaneous seizures in rats with post-traumatic epilepsy. J Neurosci Methods.

[CR2] Apostolova LG, Green AE, Babakchanian S, Hwang KS, Chou Y-Y, Toga AW, Thompson PM (2012). Hippocampal atrophy and ventricular enlargement in normal aging, mild cognitive impairment (MCI), and Alzheimer disease. Alzheimer Dis Assoc Disord.

[CR3] Boespflug EL, Iliff JJ (2018). The emerging relationship between interstitial fluid-cerebrospinal fluid exchange, amyloid-β, and sleep. Biol Psychiat.

[CR4] Bramlett HM, Dalton Dietrich W (2002). Quantitative structural changes in white and gray matter 1 year following traumatic brain injury in rats. Acta Neuropathol.

[CR5] Brezova V, Moen KG, Skandsen T, Vik A, Brewer JB, Salvesen O, Håberg AK (2014). Prospective longitudinal MRI study of brain volumes and diffusion changes during the first year after moderate to severe traumatic brain injury. NeuroImage Clinical.

[CR6] Chen L-J, Wang Y-J, Chen J-R, Tseng G-F (2017). Hydrocephalus compacted cortex and hippocampus and altered their output neurons in association with spatial learning and memory deficits in rats. Brain Pathol (Zurich, Switzerland).

[CR7] Clausen F, Hillered L (2005). Intracranial pressure changes during fluid percussion, controlled cortical impact and weight drop injury in rats. Acta Neurochirurgica.

[CR8] Dadas A, Janigro D (2019). Breakdown of blood brain barrier as a mechanism of post-traumatic epilepsy. Neurobiol Dis.

[CR9] Damkier HH, Brown PD, Praetorius J (2013). Cerebrospinal fluid secretion by the choroid plexus. Physiol Rev.

[CR10] Daou B, Klinge P, Tjoumakaris S, Rosenwasser RH, Jabbour P (2016). Revisiting secondary normal pressure hydrocephalus: does it exist? A review. Neurosurg Focus.

[CR11] Ding GL, Chopp M, Poulsen DJ, Li L, Changsheng Qu, Li Q, Nejad-Davarani SP (2013). MRI of neuronal recovery after low-dose methamphetamine treatment of traumatic brain injury in rats. PLoS One.

[CR12] Eide PK, Valnes LM, Pripp AH, Mardal K-A, Ringstad G (2020). Delayed clearance of cerebrospinal fluid tracer from choroid plexus in idiopathic normal pressure hydrocephalus. J Cereb Blood Flow Metab.

[CR13] Ge R, Tornero D, Hirota M, Monni E, Laterza C, Lindvall O, Kokaia Z (2017). Choroid plexus-cerebrospinal fluid route for monocyte-derived macrophages after stroke. J Neuroinflammation.

[CR14] Glushakova OY, Glushakov AV, Yang L, Hayes RL, Valadka AB (2020). Intracranial pressure monitoring in experimental traumatic brain injury: implications for clinical management. J Neurotrauma.

[CR15] Henning EC, Ruetzler CA, Gaudinski MR, Hu T-C, Latour LL, Hallenbeck JM, Warach S (2009). Feridex preloading permits tracking of CNS-resident macrophages after transient middle cerebral artery occlusion. J Cereb Blood Flow Metab.

[CR16] Hladky SB, Barrand MA (2016). Fluid and ion transfer across the blood-brain and blood-cerebrospinal fluid barriers; a comparative account of mechanisms and roles. Fluids Barriers CNS.

[CR17] Hu Q, Vakhmjanin A, Li Bo, Tang J, Zhang JH (2014). Hyperbaric oxygen therapy fails to reduce hydrocephalus formation following subarachnoid hemorrhage in rats. Med Gas Res.

[CR18] Hubert V, Chauveau F, Dumot C, Ong E, Berner L-P, Canet-Soulas E, Ghersi-Egea J-F, Wiart M (2019). Clinical imaging of choroid plexus in health and in brain disorders: a mini-review. Front Mol Neurosci.

[CR19] Jessen NA, Munk ASF, Lundgaard I, Nedergaard M (2015). The glymphatic system: a beginner’s guide. Neurochem Res.

[CR20] Kaur C, Singh J, Lim MK, Ng BL, Yap EP, Ling EA (1996). Studies of the choroid plexus and its associated epiplexus cells in the lateral ventricles of rats following an exposure to a single non-penetrative blast. Arch Histol Cytol.

[CR21] Kharatishvil I, R Immonen, O Gröhn, and A Pitkänen (2007) ‘Quantitative diffusion MRI of hippocampus as a surrogate marker for post-traumatic epileptogenesis’. Brain. 130 (Pt 12): 3155–68. 10.1093/brain/awm268. 10.1093/brain/awm268 18055492

[CR22] Kharatishvili I, Nissinen JP, McIntosh TK, Pitkänen A (2006). A model of posttraumatic epilepsy induced by lateral fluid-percussion brain injury in rats. Neuroscience.

[CR23] Kowalski RG, Weintraub AH, Rubin BA, Gerber DJ, Olsen AJ (2018). Impact of timing of ventriculoperitoneal shunt placement on outcome in posttraumatic hydrocephalus. J Neurosurg..

[CR24] Kress BT, Iliff JJ, Xia M, Wang M, Wei HS, Zeppenfeld D, Xie L (2014). Impairment of paravascular clearance pathways in the aging brain. Ann Neurol.

[CR25] Lapinlampi N, Melin E, Aronica E, Bankstahl JP, Becker A, Bernard C, Gorter JA (2017). Common data elements and data management: remedy to cure underpowered preclinical studies. Epilepsy Res.

[CR26] Liu S, Grigoryan MM, Vasilevko V, Sumbria RK, Paganini-Hill A, Cribbs DH, Fisher MJ (2014). Comparative analysis of H and E and prussian blue staining in a mouse model of cerebral microbleeds. J Histochem Cytochem.

[CR27] Luikku AJ, Hall A, Nerg O, Koivisto AM, Hiltunen M, Helisalmi S, Herukka S-K (2019). Predicting development of Alzheimer’s disease in patients with shunted idiopathic normal pressure hydrocephalus. J Alzheimer’s Dis.

[CR28] Malm J, Graff-Radford NR, Ishikawa M, Kristensen Bo, Leinonen V, Mori E, Owler BK, Tullberg M, Williams MA, Relkin NR (2013). Influence of comorbidities in idiopathic normal pressure hydrocephalus-research and clinical care. A report of the ISHCSF task force on comorbidities in INPH. Fluids Barriers CNS.

[CR29] Marchi N, Banjara M, Janigro D (2016). Blood-brain barrier, bulk flow, and interstitial clearance in epilepsy. J Neurosci Methods.

[CR30] Marmarou A, Foda MA, Bandoh K, Yoshihara M, Yamamoto T, Tsuji O, Zasler N, Ward JD, Young HF (1996). Posttraumatic ventriculomegaly: hydrocephalus or atrophy? A new approach for diagnosis using CSF dynamics. J Neurosurg.

[CR31] Mazzini L, Campini R, Angelino E, Rognone F, Pastore I, Oliveri G (2003). Posttraumatic hydrocephalus: a clinical, neuroradiologic, and neuropsychologic assessment of long-term outcome. Arch Phys Med Rehabil.

[CR32] Millward JM, Ariza A, de Schellenberger D, Berndt L-V, Schellenberger E, Waiczies S, Taupitz M, Kobayashi Y, Wagner S, Infante-Duarte C (2019). Application of europium-doped very small iron oxide nanoparticles to visualize neuroinflammation with MRI and fluorescence microscopy. Neuroscience.

[CR33] Millward JM, Schnorr J, Taupitz M, Wagner S, Wuerfel JT, Infante-Duarte C (2013). Iron oxide magnetic nanoparticles highlight early involvement of the choroid plexus in central nervous system inflammation. ASN Neuro.

[CR34] Mishra M, Singh R, Mukherjee S, Sharma D (2013). Dehydroepiandrosterone’s antiepileptic action in FeCl3-induced epileptogenesis involves upregulation of glutamate transporters. Epilepsy Res.

[CR35] Morris R (1984). Developments of a water-maze procedure for studying spatial learning in the rat. J Neurosci Methods.

[CR36] Murtha LA, Yang Q, Parsons MW, Levi CR, Beard DJ, Spratt NJ, McLeod DD (2014). Cerebrospinal fluid is drained primarily via the spinal canal and olfactory route in young and aged spontaneously hypertensive rats. Fluids Barriers CNS.

[CR37] Nestor SM, R Rupsingh, M Borrie, M Smith, V Accomazzi, JL Wells, J Fogarty, R Bartha, Alzheimer’s Disease Neuroimaging Initiative (2008) Ventricular enlargement as a possible measure of alzheimer’s disease progression validated using the Alzheimer’s disease neuroimaging initiative database. Brain. 131 (Pt 9): 2443–54. 10.1093/brain/awn146. 10.1093/brain/awn146 PMC2724905 18669512

[CR38] Olopade FE, Shokunbi MT, Sirén A-L (2012). The Relationship between ventricular dilatation, neuropathological and neurobehavioural changes in hydrocephalic rats. Fluids Barriers CNS.

[CR39] Piatt JH, Carlson CV (1996). Hydrocephalus and epilepsy: an actuarial analysis. Neurosurgery.

[CR40] Qi Z, Zhang H, Chuhua Fu, Liu X, Chen Bo, Dang Y, Chen H, Liu L (2017). Prolonged hydrocephalus induced by intraventricular hemorrhage in rats is reduced by curcumin therapy. Neurosci Lett.

[CR41] Ren Z, Iliff JJ, Yang L, Yang J, Chen X, Chen MJ, Giese RN, Wang B, Shi X, Nedergaard M (2013). “Hit & Run” model of closed-skull traumatic brain injury (TBI) reveals complex patterns of post-traumatic AQP4 dysregulation. J Cereb Blood Flow Metab.

[CR42] Ringstad G, Svein Are Sirirud Vatnehol, and Per Kristian Eide (2017) glymphatic MRI in idiopathic normal pressure hydrocephalus. Brain. 140(10): 2691–2705. 10.1093/brain/awx191. 10.1093/brain/awx191 PMC5841149 28969373

[CR43] Sharma V, Prakash Babu P, Singh A, Singh S, Singh R (2007). Iron-induced experimental cortical seizures: electroencephalographic mapping of seizure spread in the subcortical brain areas. Seizure.

[CR44] Stadler A, Schima W, Ba-Ssalamah A, Kettenbach J, Eisenhuber E (2007). Artifacts in body MR imaging: their appearance and how to eliminate them. Eur Radiol.

[CR45] Sullan MJ, Asken BM, Jaffee MS, DeKosky ST, Bauer RM (2018). Glymphatic system disruption as a mediator of brain trauma and chronic traumatic encephalopathy. Neurosci Biobehav Rev.

[CR46] Szmydynger-Chodobska J, Strazielle N, Gandy JR, Keefe TH, Zink BJ, Ghersi-Egea J-F, Chodobski A (2012). Posttraumatic invasion of monocytes across the blood-cerebrospinal fluid barrier. J Cereb Blood Flow Metab.

[CR47] Szmydynger-Chodobska J, Strazielle N, Zink BJ, Ghersi-Egea J-F, Chodobski A (2009). The role of the choroid plexus in neutrophil invasion after traumatic brain injury. J Cereb Blood Flow Metab.

[CR48] Turtzo LC, Budde MD, Gold EM, Lewis BK, Janes L, Yarnell A, Grunberg NE, Watson W, Frank JA (2013). The evolution of traumatic brain injury in a rat focal contusion model. NMR Biomed.

[CR49] Wang Y, Li Z, Zhang X, Chen Z, Li D, Chen W, Jiamei Gu, Sun D, Rong T, Kwan P (2021). Development and validation of a clinical score to predict late seizures after intracerebral hemorrhage in Chinese. Epilepsy Res.

[CR50] Weintraub AH, Gerber DJ, Kowalski RG (2017). Posttraumatic hydrocephalus as a confounding influence on brain injury rehabilitation: incidence, clinical characteristics, and outcomes. Arch Phys Med Rehabil.

[CR51] Wiart M, Davoust N, Pialat J-B, Desestret V, Moucharrafie S, Moucharaffie S, Cho T-H (2007). MRI monitoring of neuroinflammation in mouse focal ischemia. Stroke.

[CR52] Willmore LJ, Rubin JJ (1981). Antiperoxidant pretreatment and iron-induced epileptiform discharges in the rat: EEG and histopathologic studies. Neurology.

[CR53] Xiang J, Routhe LJ, Andrew Wilkinson D, Hua Ya, Moos T, Xi G, Keep RF (2017). The choroid plexus as a site of damage in hemorrhagic and ischemic stroke and its role in responding to injury. Fluids Barriers CNS.

[CR54] Zhang C, Wang X, Wang Y, Zhang J-G, Wenhan Hu, Ge M, Zhang K, Shao X (2014). Risk factors for post-stroke seizures: a systematic review and meta-analysis. Epilepsy Res.

[CR55] Zhao J, Chen Z, Xi G, Keep RF, Hua Ya (2014). Deferoxamine attenuates acute hydrocephalus after traumatic brain injury in rats. Transl Stroke Res.

