# Supplementary material for: Post-injury ventricular enlargement associates with iron in choroid plexus but not with seizure susceptibility nor lesion atrophy—6-month MRI follow-up after experimental traumatic brain injury
Source: Brain Struct Funct. 2021 Nov 10;227(1):145–58. doi: 10.1007/s00429-021-02395-5 (PMC8741668; doi:10.1007/s00429-021-02395-5)
Supplement: Supplementary file 1 — Supplementary file1 (PDF 866 KB) [file 429_2021_2395_MOESM1_ESM.pdf]

Supplementary data

Journal: Brain structure and Function

Post-injury ventricular enlargement associates with iron in choroid plexus but not with seizure susceptibility nor lesion atrophy - 6 months MRI follow-up after experimental traumatic brain injury

Amna Yasmin[1], Asla Pitkänen[1], Pedro Andrade[1], Tomi Paananen[1], Olli Gröhn[1] and Riikka Immonen[1,*]

[1]A.I.Virtanen Institute for Molecular Sciences, University of Eastern Finland, P.O.Box 1627, Kuopio, Finland

***Correspondence:**

Riikka Immonen
riikka.immonen@uef.fi
ORCID 0000-0002-9855-7098

This supplementary material describes:

1) the *temporal pattern* of the choroid plexus bleeds in the lateral fluid percussion injury (FPI) rat model from the day 2 post-injury onwards (a case study with multi gradient echo MRI data)
2) the iron findings along the internal cerebral vein that connects to the choroidal bleed location (study cohort and a case study with multi gradient echo MRI data)
3) how the chronic expansion of the ventricles correlate with the chronic cortical hypoperfusion, that is, the cerebral blood flow decrease in the adjacent cortical parenchyma 6 months post-injury

Acute choroid plexus bleeds at 2 d post-injury and evolution of choroid plexus iron in MRI 2 d, 9 d, 30 d and 3 months post-injury, a case study

We extended our examination of the temporal evolution of choroid plexus bleeds to the acute phase after FPI. Case study below (**Suppl. Fig1**) shows that choroid plexus bleed is detected already 2 d post-injury by *in vivo* MRI, and that the radiological appearance of the choroidal hypointensity stabilizes after 30 d.

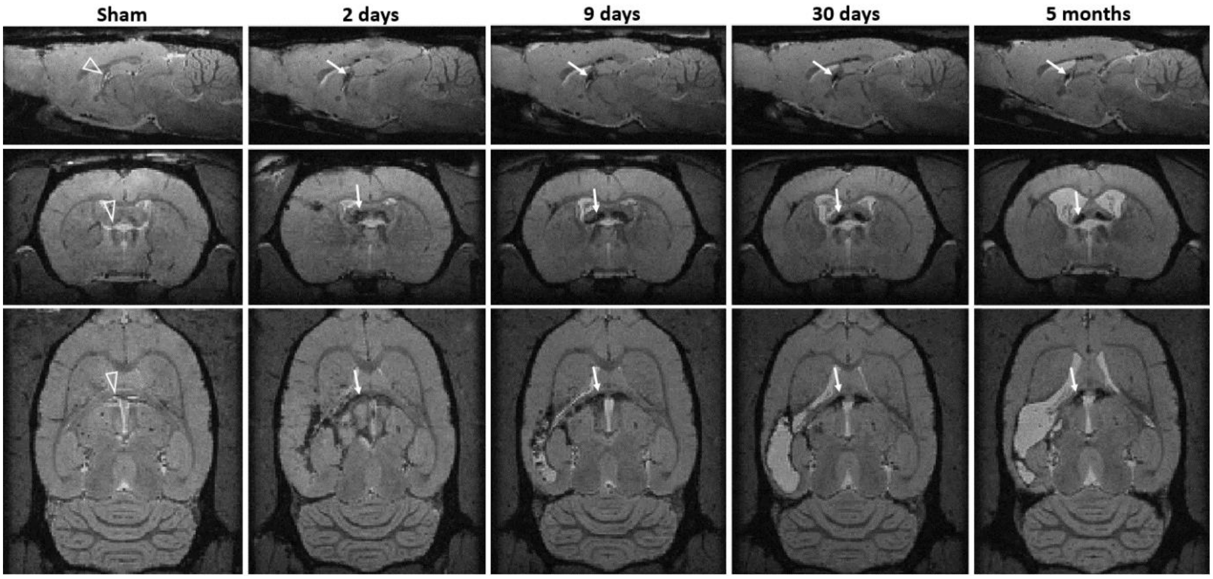

Suppl. Fig. 1 ***Evolution of choroid plexus injury in MRI – a case study*****.** Sagittal (upper row), coronal (middle) and horizontal (bottom) view of choroid plexus iron signal (black signal void, white arrow) characteristics over time. Column 1 (left): a sham-operated experimental control rat. Columns 2-5: evolution of choroid plexus injury over time in a representative rat with lateral fluid-percussion injury (FPI). At 2 d after lateral FPI (column 2), the iron signal in the choroid plexus appeared more diffuse/dispersed than at later time points. By 9 d (column 3), the iron signal had become more intense and compact, suggesting iron aggregation. Over the next 5 months, the iron signal remained similar, showing no signs of clearance or further accumulation. Note the parallel expansion of the ipsilateral ventricle which precedes the contralateral ventricle enlargement. Data are T2*/T1w multigradient echo 3D images with 160 μm^3 isotropic resolution. Similar evolution pattern is seen in larger FPI cohort as well (EpiBioS4Rx cohort, data not shown).

Iron along the internal cerebral vein

While screening the sagittal images of the 3D FISP, we observed iron also along the internal cerebral vein (straight sinus) that runs to choroid plexus in sagittal midline. Internal cerebral veins are paired paramedian veins that are formed at the foramen of Monro by the confluence of the choroidal vein and that thus are draining the choroid plexus of the lateral ventricle, draining the site of the original bleeds. This observation raised a question, whether the finding presented a downstream effect for the choroidal bleeds or a separate mechanical injury. We found that while 18/20 TBI rats had choroidal iron, only 4 of the 18 also had iron along the internal cerebral vein (**Suppl. Fig 2**.) Importantly, these 4 cases were not the ones with the most massive choroid plexus iron accumulation. This suggested that iron in internal cerebral vein was a sign of a separate injury from that of choroid plexus hemorrhages, and could present another contributor to the process leading the chronic ventricle enlargement.

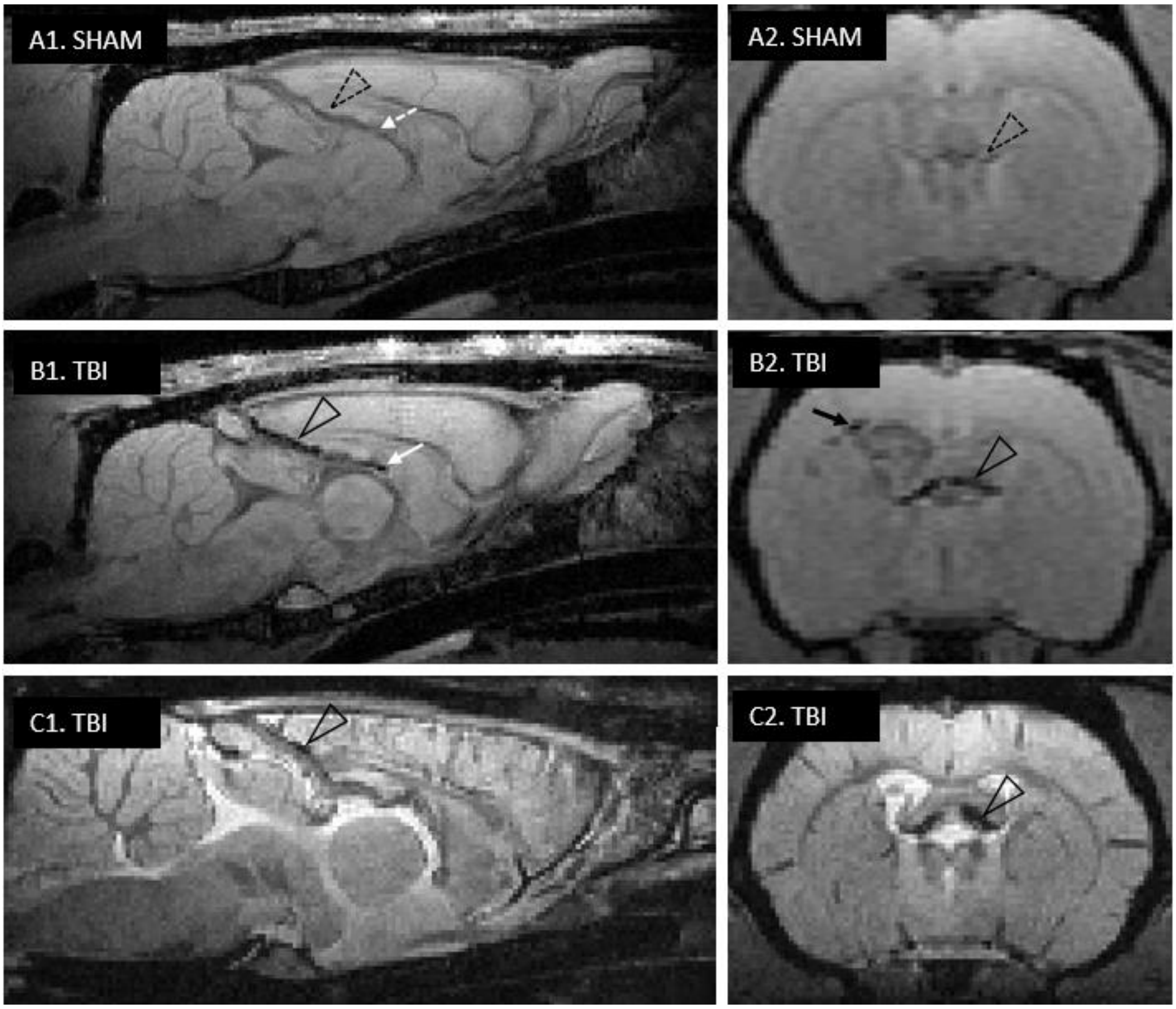

Suppl. Fig. 2 *Internal cerebral vein iron*. A-B FISP images depicting post TBI iron along the internal cerebral vein (left column, sagittal view) and in the choroid plexus of the same animal (right column, coronal view). Sham animals had no iron along internal cerebral vein (**A1**, dashed arrow head), or in choroid plexus (**A2**, dashed arrow head). TBI animals showed iron in choroid plexus (**B2**, arrow head) in majority of cases, but only 4/18 TBIs with choroidal bleeds also had iron along internal cerebral vein (**B1**, arrow head). **C**. T2*/T1w images with a contrast that shows CSF bright aids to identify the vessel structure and the iron in respect to the CSF space. White arrow (in **A1** and **B1**) depicts the location where the two lateral vascular branches arrive from left and right. Black arrow (**B2**) depicts iron from white matter microbleed. Note, how massive the choroidal iron deposits are in comparison to that. Time point is one month post-injury. Bottom row T2*/T1w multi gradient echo 3D images with 160 μm^3 isotropic resolution. Structures in MRI were identified based on their correspondence with the sagittal brain-with-meninges-sections according to Hashimoto (1985). In FISP (**panel**

B1) the iron pops out clearly against the flat background, while the contrast in T2*/T1w images with bright CSF (**panel C1**) aids to distinguish the vein in the roof of 3rd ventricle running towards pineal recess (CSF bright, vein dark). The iron residues along vena cerebri interna could also be a downstream effect as it receives blood from choroid plexus, but it was not a systemic finding.

Expansion of the ventricles correlates with the decreased adjacent cortical blood flow

We studied if the ventricle volume in the end of the follow-up was associated with the CBF deficits in the surrounding cortical parenchyma. Cortical vascular pulsation deficit could hinder the CSF circulation and thus be one driver for the ventricular expansion. Analysis focused on the contralateral ventricle expansions and contralateral cortex to avoid confounding factors of the perilesional area. At 6 months after lateral FPI, the cortical CBF' in the contralateral primary somatosensory cortex (S1BF) was decreased in rats with lateral FPI (77 ± 15 ml/100g/min) as compared to that in sham-operated experimental controls (98 ± 7 ml/100g/min, p<0.05). A non-significant trend was also observed in the more frontally located S1 area (TBI 86 ± 20 ml/100g/min vs. sham 99 ± 12 ml/100g/min). Pearson correlation analysis indicated that the lower the CBF' at 6 months, the larger the contralateral ventricle volume at 6 months in the S1BF (r=-0.633, p<0.01). Similar trend was observed also in S1 but the significance did not tolerate Bonferroni correction for multiple comparisons (r=-0.447, p>0.05) (**Suppl. Fig 3**). The contralateral cortical CBF deficit did not correlate with seizure susceptibility in the PTZ-test.

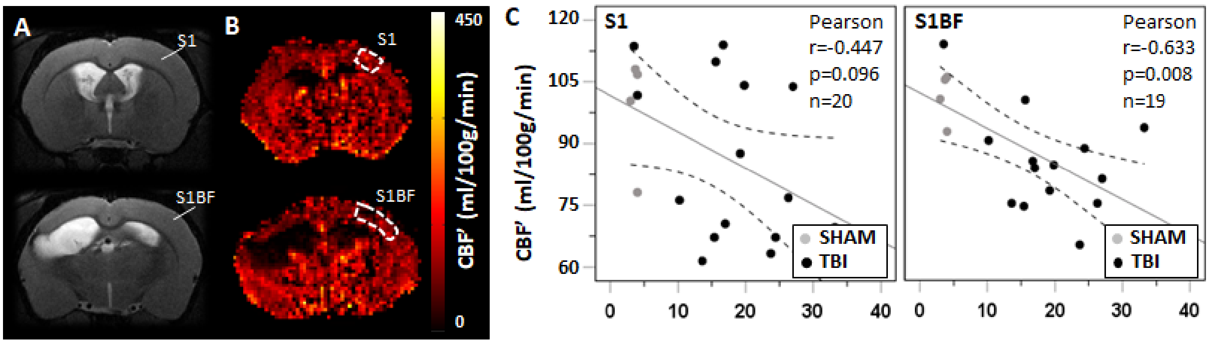

Suppl. Fig. 3 ***Cortical blood perfusion deficit correlates with ventricle volume at 6 months postinjury*. (A)** T2 weighted images and **(B)** cerebral blood flow (CBF)

maps at corresponding level. CBF maps were measured by arterial spin-labeling MRI at 6 months post FPI to assess the blood perfusion. Cortical region of interests (dashed outlines) included the primary somatosensory cortex (S1) at -0.5 mm from bregma (top row) and the S1 barrel field (S1BF) at -2.0 mm from bregma (bottom row; slice thickness of CBF maps 1.5 mm) adjacent to the progressively enlarging contralateral ventricle. **(C)** CBF in contralateral S1BF correlated with the contralateral ventricle volume at 6 months post-TBI ($p<0.01$). No correlation was found in S1. Statistical analysis was done using Pearson correlation with Bonferroni for multiple comparisons, Scatter plots show the linear regression line with 95% confidence intervals. Note the method description below for CBF': absolute CBF values (designated as CBF') have systematic bias that does not compromise the correlation assessment.

Method: Cerebral blood flow (CBF) by arterial spin labeling

Blood perfusion MRI was performed at 6 months post-injury in order to detect potential cortical hypoperfusion adjacent to the contralateral ventricle. Pseudo-continuous arterial spin labeling (CASL) was used to map the CBF in multiple coronal slices. CASL used a train of block pulses as label (180 inversion of inflowing spins) at neck level (-20mm offset), and Double Adiabatic Inversion (Werner et al. 2005) of flowing spins as control (2*180 flip -> total flip 0) in the same -20 mm offset location. Label time was 1800 msec, post label time 630 msec, label gradient 596 Hz/mm, EPI multi-slice readout (all slices collected tightly at the beginning of TR, thus 'delay between volumes' parameter set was maximum of 3163 msec, slices collected reversed sequential order: 4, 3, 2, 1, 0), 1 segment, TR 6 s, TE 38 msec, SW 250 kHz, FOV 36x36 mm, 128x128, 5 slices 1.5 mm thick, gap 0, fat suppression, 24 repetitions of control-label pair. Scan time was 4 min 48 sec. Lambda 0.9 ml/g, and constant $T1_{blood}$ of 2.1 sec was used in the CBF calculation (Zhang et al. 2016).

After movement correction, the signal difference between control and label images (*i.e.,* the asl-map) was calculated by pair-wise subtraction, and thereafter, averaged over repetitions. CBF as ml/100g/min was calculated as CBF=[100*60*lambda*asl-map]/[$T1_{blood}$*2*control_mean]. The control-label signal difference was only 3.5-4% resulting in the values in sham controls to be 1/3rd of that reported in the literature (Larkin et al. 2019). The suboptimal labeling efficacy was due

to suboptimal RF-coil configuration available combined with the large size of the rats (>500g): the length of the transmit volume coil was 12 cm (total length 12 cm, sensitive volume approximately 8 cm), and thus the labeling pulse was performed in the periphery of the sensitive volume of the RF-coil leading to lower and somewhat inhomegonous B1. Therefore, to correct for the systematic bias, we report values here (designated as CBF') where the suboptimal labeling efficiency is accounted for by employing a correction factor of 3 on all CSF values. Two cortical ROIs adjacent to the enlarged ventricles were manually drawn for CBF analysis (see **Suppl. Fig 3A-B**) on primary sensory cortex (S1) and S1 barrel field (S1BF).

References

Larkin, James R., Manon A. Simard, Alexandre A. Khrapitchev, James A. Meakin, Thomas W. Okell, Martin Craig, Kevin J. Ray, Peter Jezzard, Michael A. Chappell, and Nicola R. Sibson. 2019. 'Quantitative Blood Flow Measurement in Rat Brain with Multiphase Arterial Spin Labelling Magnetic Resonance Imaging'. *Journal of Cerebral Blood Flow and Metabolism: Official Journal of the International Society of Cerebral Blood Flow and Metabolism* 39 (8): 1557–69. https://doi.org/10.1177/0271678X18756218.

Werner, Richard, David G. Norris, Karsten Alfke, H. Maximilian Mehdorn, and Olav Jansen. 2005. 'Improving the Amplitude-Modulated Control Experiment for Multislice Continuous Arterial Spin Labeling'. *Magnetic Resonance in Medicine* 53 (5): 1096–1102. https://doi.org/10.1002/mrm.20443.

Zhang, X., E. T. Petersen, E. Ghariq, J. B. De Vis, A. G. Webb, W. M. Teeuwisse, J. Hendrikse, and M. J. P. van Osch. 2013. 'In Vivo Blood T(1) Measurements at 1.5 T, 3 T, and 7 T'. *Magnetic Resonance in Medicine* 70 (4): 1082–86. https://doi.org/10.1002/mrm.24550.
